# Eduardo E. Benarroch. Neuroscience for Clinicians: Basic Processes, Circuits, Disease Mechanisms, and Therapeutic Implications Oxford University Press; 2021. 832 pages. ISBN: 9780190948894 (hardcover)

**DOI:** 10.3325/cmj.2021.62.643

**Published:** 2021-12

**Authors:** Dora Sedmak

Medical fields: neuroscience, neurology, neurosurgery

Target audience: As the title suggests, this book is a great read for anyone interested in neuro-related fields, as well as neurologists, neurosurgeons, doctoral students, and all practitioners in search of a comprehensive but not an excessively detailed read.

Purpose: Due to rapid technological advances, novel approaches to the science and understanding of the human nervous system are being constantly developed. Despite this, the primary role of the clinician remains essentially the same. This book aims to bridge the ever-changing gap between basic and clinical neuroscience.

Content: The book consists of 6 sections and 42 chapters that cover the most important concepts in neuroscience, from its basics to the novel neuroscientific approaches and the pathophysiology of disorders. The first chapters mainly focus on molecular concepts, while the last ones cover the integration of the nervous system on different levels (networks, plasticity, control etc). Each chapter begins with a brief introduction, followed by supporting figures, and ends with key messages accompanied by a list of relevant references.

The first section gives an overview of molecular and cellular mechanisms such as signaling, cell membranes, cell death, and regeneration. Section II explains the communication between neurons, and provides information on ion channels, receptors, neurotransmission, and neuromodulation. In Section III, glial cells and microenvironment are covered. While section IV discusses neuronal interaction in cortical and subcortical circuits, motor, sensory and behavioral circuits, as well as circadian rhythms, section V covers pain, nociception, autonomic control, and homeostasis. The last section deals with the complex circuits such as language, memory, emotional and social cognition, and executive control.

Highlights: The book is an easy-to-read recapitulation of basic neuroscience concepts and mechanisms important for the development of disorders. It can almost be considered a hands-on manual serving as a quick reminder of where, how, and why some disorders develop. The content is reader-friendly, with a brief introduction and key messages listed at the end of each chapter.

Limitations: In order to benefit from this book, the reader needs to be familiar with the basic principles of the functioning of the nervous system on both molecular and anatomical levels. The book is not intended for beginners, but it successfully highlights the most important basic and clinical concepts in neuroscience. Hard copy is the preferable format, because numerous illustrations cannot by easily viewed when the book is accessed via the publisher's digital platform.

Related reading: The end of each chapter offers a list of references that would be of interest to the reader. There are similar books on the market, such as a less comprehensive title, more suitable for a reader with no background in the field: Simpkins CA, Simpkins, AM. Neuroscience for clinicians: Evidence, models and practice. (2013) Springer. https://doi.org/10.1007/9781461448426

Commentary: I would recommend the book to anyone looking for a comprehensive but not excessively detailed neuroscience recap with up-to-date clinical approaches.

